# Anti-SARS-CoV-2 antibody levels predict outcome in COVID-19 patients with type 2 diabetes: a prospective cohort study

**DOI:** 10.1038/s41598-023-45700-4

**Published:** 2023-10-26

**Authors:** Sylvia Mink, Christoph H. Saely, Andreas Leiherer, Matthias Frick, Thomas Plattner, Heinz Drexel, Peter Fraunberger

**Affiliations:** 1 Central Medical Laboratories, Carinagasse 41, 6800 Feldkirch, Vorarlberg Austria; 2 Private University in the Principality of Liechtenstein, Triesen, Principality of Liechtenstein; 3 VIVIT Institute, Academic Teaching Hospital Feldkirch, Feldkirch, Austria; 4 Department of Internal Medicine, Academic Teaching Hospital Feldkirch, Feldkirch, Austria; 5 Drexel University College of Medicine, Philadelphia, PA USA

**Keywords:** Predictive markers, Prognostic markers, Viral infection, Diabetes

## Abstract

Patients with type 2 diabetes (T2D) constitute one of the most vulnerable subgroups in COVID-19. Despite high vaccination rates, a correlate of protection to advise vaccination strategies for novel SARS-CoV-2 variants of concern and lower mortality in this high-risk group is still missing. It is further unclear what antibody levels provide protection and whether pre-existing organ damage affects this threshold. To address these gaps, we conducted a prospective multicenter cohort study on 1152 patients with COVID-19 from five hospitals. Patients were classified by diabetes and vaccination status. Anti-SARS-CoV-2-spike-antibodies, creatinine and NTproBNP were measured on hospital admission. Pre-specified endpoints were all-cause in-hospital-mortality, ICU admission, endotracheal intubation, and oxygen administration. Propensity score matching was applied to increase comparability. We observed significantly lower anti-SARS-CoV-2-spike-antibodies in diabetic non-survivors compared to survivors (mean, 95% CI 351BAU/ml, 106–595 vs. 1123, 968–1279, *p* < 0.001). Mortality risk increased two-fold with each standard deviation-decrease of antibody levels (aHR 1.988, 95% CI 1.229–3.215, *p* = 0.005). T2D patients requiring oxygen administration, endotracheal intubation and ICU admission had significantly lower antibody levels than those who did not (*p* < 0.001, *p* = 0.046, *p* = 0.011). While T2D patients had significantly worse outcomes than non-diabetic patients, the differences were less pronounced compared to propensity-score-matched non-diabetic patients. Anti-SARS-CoV-2 spike antibodies on hospital admission are inversely associated with oxygen administration, endotracheal intubation, intensive care and in-hospital mortality in diabetic COVID-19 patients. Pre-existing comorbidities may have a greater impact on outcome than diabetes status alone.

## Introduction

Patients with type 2 diabetes (T2D) are among the most severely affected subgroups in COVID-19^1–4^. Globally, diabetes accounts for approximately 9.5% of severe cases and 16.8% of COVID-19 related deaths^1^. In comparison to the general population, patients with diabetes are reported to have 1.6 times higher odds of being hospitalized, 1.9 times higher odds of requiring intensive care and 1.4 times higher odds of death^2^.

Several factors have been suggested to contribute to increased disease severity and higher mortality rates, including hypercoagulation through overexpression of prothrombotic factors, upregulation of inflammatory cytokines, reduced respiratory function, endothelial dysfunction, aggravation of preexisting insulin-resistance and association with other comorbidities such as chronic obstructive pulmonary disease and renal disease^5–7^.

A recent metaanalysis^8^ comprising over 11,000 patients with diabetes reported an overall pooled vaccine acceptance rate of 76.1%. Despite the high degree of vaccination coverage, a correlate of protection to advise vaccination strategies^9^ for novel SARS-CoV-2 variants of concern and mitigate elevated mortality risks in this high-risk subgroup is still missing. We previously reported that anti-SARS-CoV-2 spike antibodies on hospital admission are inversely associated with in-hospital mortality^10^.

Given the elevated risks of severe disease and COVID-19 mortality in patients with type 2 diabetes, understanding the connection between anti-SARS-CoV-2 antibodies and outcome is critical for identifying individuals at high risk of severe courses and to inform future strategies for booster vaccinations in this high-risk patient group.

In this prospective, multicenter cohort study, we evaluate the usefulness of anti-SARS-CoV-2 spike antibodies as a correlate of protection in hospitalized, T2D patients. We further stratified patients by cardial and renal impairments to assess the influence of preexisting organ damage on anti-SARS-COV2 antibodies as a potential correlate of protection.

## Results

### Participants

**Figure 1:**
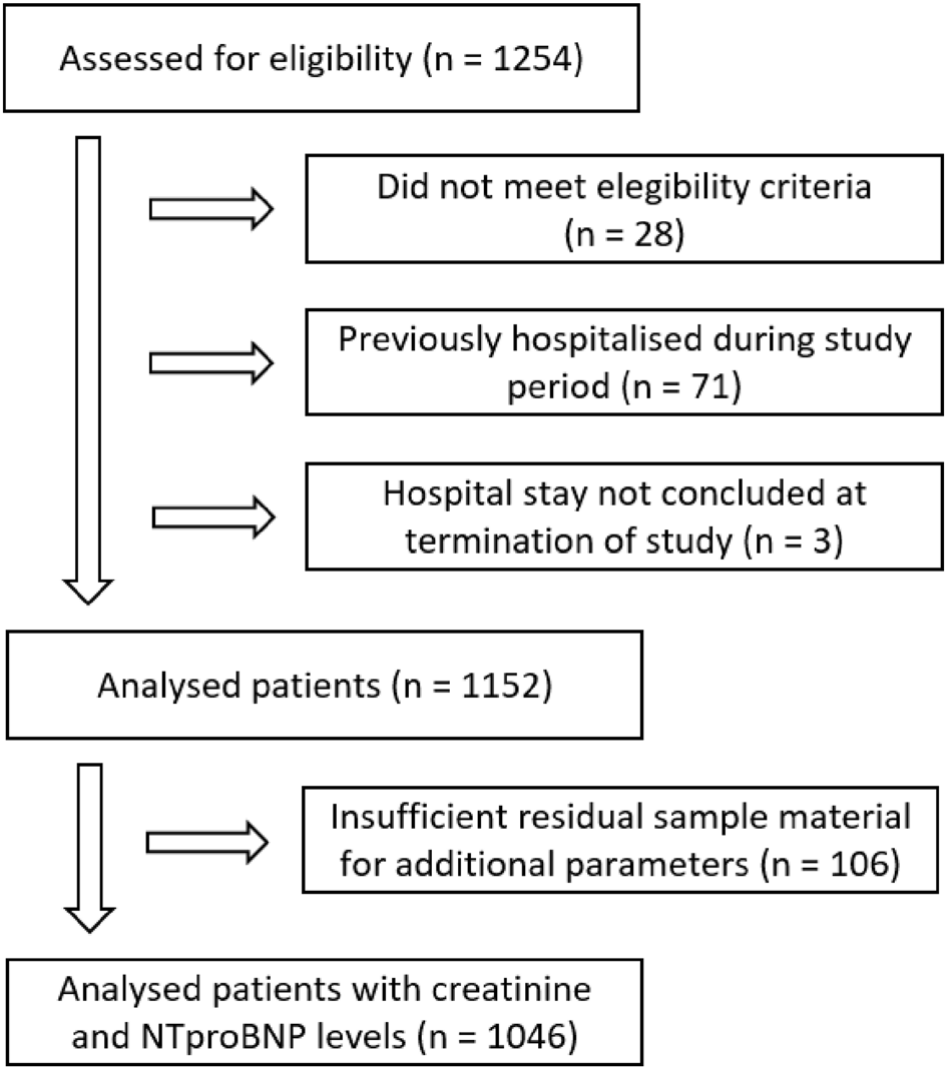
Patient flow diagram.

Between August 1st, 2021 and April 10th, 2022 a total of 1254 hospitalized patients were assessed for eligibility at five hospitals. Of these, 1152 patients were enrolled in the study and subsequently analysed. Anti-SARS-CoV-2-spike antibody levels were measured in all 1152 patients. Additional parameters such as creatinine and NT-proBNP could only be measured in 1046 patients due to insufficient residual sample material. Patient flow is outlined in Fig. 1.

Of the study population, 118 patients did not survive, 165 patients were admitted to an intensive care unit, 47 patients required endotracheal intubation and 587 patients required oxygen administration. 275 patients either had a history of diabetes or were diagnosed during their hospital stay. Table 1 shows patient characteristics by diabetes status for diabetic, non-diabetic and matched, non-diabetic patients. Table 2 outlines patient characteristics and outcomes by vaccination status.

**Table 1:** Patient characteristics and outcomes for type 2 diabetes patients, non-diabetic and matched, non-diabetic patients.

	Whole cohort n = 1152	DM n = 275	No DM n = 877	*p*-Value	No DM, matched n = 232	*p*-Value (DM vs. no DM, matched)
Age (years)	66.8 ± 20.3	75.1 ± 12.2	64.2 ± 21.6	** < 0.001**	71.8 ± 16.3	0.101
Male gender (%)	53.2	53.5	53.1	0.926	50.0	0.438
BMI (kg/m^2^)	27.1 ± 6.5	29.1 ± 6.2	26.4 ± 6.4	** < 0**.**001**	28.0 ± 6.3	**0.020**
DM (%)	23.9	/	/	/	/	/
Hypertension (%)	50.5	77.5	42.1	** < 0.001**	64.7	**0.001**
CAD (%)	21.6	36.4	17.0	** < 0.001**	29.3	0.093
Heart failure (%)	7.2	13.1	5.4	** < 0.001**	8.6	0.110
COPD (%)	9.6	14.9	8.0	** < 0.001**	13.4	0.619
Asthma (%)	2.4	1.5	2.7	0.228	2.2	0.552
Renal disease (%)	22.9	39.3	18.0	** < 0.001**	29.3	**0.018**
Stroke/TIA/CVD (%)	11.7	18.9	9.5	** < 0.001**	16.4	0.458
Mortality (%)	10.2	16.0	8.4	** < 0.001**	13.4	0.405
ICU (%)	14.3	19.6	12.7	**0.004**	18.5	0.753
Intubation (%)	4.1	5.1	3.8	0.337	7.8	0.218
Oxygen req. (%)	51.0	65.0	46.6	** < 0.001**	59.9	0.242
CT value	21.3 ± 6.6	20.8 ± 6.6	21.4 ± 6.5	0.085	20.6 ± 6.3	0.908
Creatinine (mg/dl)	1.25 ± 1.07	1.55 ± 1.25	1.15 ± 0.99	** < 0.001**	1.16 ± 0.59	** < 0.001**
NT-proBNP (pg/ml)	1971 ± 5297	2876 ± 6202	1689 ± 4953	** < 0.001**	1973 ± 5315	**0.033**
Spike ab (BAU/ml)	946 ± 1151	999 ± 1175	930 ± 1144	0.263	828 ± 1118	0.098

Quantitative results are given as means ± standard deviation. *BMI* body mass index, *DM* diabetes mellitus, *CAD* coronary artery disease, *COPD* chronic obstructive pulmonary disease, *TIA* transient ischemic attack, *CVD* cerebrovascular disease, *ICU* intensive care unit, intubation- endotracheal intubation, oxygen req—oxygen requirement, *CT* cycle threshold, *spike ab—anti-SARS-CoV-2* spike antibodies, bold print- statistically significant.

**Table 2:** Patient characteristics and outcomes for vaccinated and non-vaccinated patients with type 2 diabetes.

	DM, vaccinated n = 172	DM, non-vaccinated n = 103	*p*-value
Age (years)	76.6 ± 10.1	72.7 ± 14.9	0.068
Male gender (%)	56.4	48.5	0.206
BMI (kg/m2)	28.6 ± 5.9	29.9 ± 6.6	0.123
DM (%)	–	–	–
Hypertension (%)	80.8	71.8	0.085
CAD (%)	40.1	30.1	0.095
Heart failure (%)	14.5	10.7	0.359
COPD (%)	18.6	8.7	**0.026**
Asthma (%)	1.7	1.0	0.604
Renal disease (%)	47.3	26.2	** < 0.001**
Stroke/TIA/CVD (%)	18.6	19.4	0.868
Mortality (%)	10.5	25.2	**0.001**
ICU (%)	14.0	29.1	**0.002**
Endotracheal intubation (%)	2.3	9.7	**0.007**
Oxygen administration (%)	57.3	77.7	** < 0.001**
CT value	20.8 ± 7.1	20.8 ± 5.8	0.710
Creatinine (mg/dl)	1.7 ± 1.5	1.3 ± 0.7	**0.027**
NT-proBNP (pg/ml)	3179 ± 6331	2357 ± 5979	**0.010**
Spike antibodies (BAU/ml)	1537 ± 1144	111 ± 485	** < 0.001**

Quantitative results are given as means ± standard deviation. *BMI* body mass index, *DM* diabetes mellitus, *CAD* coronary artery disease, *COPD* chronic obstructive pulmonary disease, *TIA* transient ischemic attack, *CVD* cerebrovascular disease, *ICU* intensive care unit, *CT* cycle threshold, *BAU* binding antibody units, bold print- statistically significant.

In comparison to non-diabetic patients, T2D patients were on average 11 years older and had significantly higher BMIs. T2D patients also had significantly higher rates of hypertension, coronary artery disease, heart failure, chronic obstructive pulmonary disease, renal diseases and cerebrovascular disease. Mortality rates, ICU admission and oxygen administration were significantly higher in T2D patients than in non-diabetic patients.

In order to increase comparability between diabetic and non-diabetic patients, we conducted propensity score matching. Compared to matched, non-diabetic patients, T2D patients still registered higher rates of mortality, ICU admission and oxygen administration, albeit the differences were no longer statistically significant.

### Anti-SARS-CoV-2 antibody levels by outcome

Anti-SARS-Cov2 spike antibody levels were significantly lower in non-survivors than in survivors across all investigated patient subgroups—including T2D patients, non-diabetic patients and matched, non-diabetic patients (binding antibody units (BAU)/ml; diabetic mean 351, 95% CI 106–595 vs. 1123, 968–1279, *p* < 0.001; non-diabetic 511, 281–742 vs. 968, 888–1048, *p* < 0.001; matched, non-diabetic 246, 0–507 vs. 920, 761–1079, *p* < 0.001). This association continued to be statistically significant after stratification of T2D patients by vaccination status (diabetic vaccinated 846, 302–1390 vs. 1618, 1438–1799, *p* = 0.006 and diabetic non-vaccinated 8, 0–21 vs. 146, 20–273, *p* = 0.014).

Similarly, diabetic, non-diabetic and matched, non-diabetic patients with lower anti-SARS-CoV-2 spike antibodies were more likely to be admitted to an intensive care unit than patients with higher levels (BAU/ml; diabetic mean 659, 363–955 vs. 1081, 923–1238, *p* = 0.011; non-diabetic 458, 283–632 vs. 998, 916–1080, *p* < 0.001; non-diabetic, matched 195, 0–397 vs. 974, 807–1139, *p* < 0.001). However, antibody levels did not differ significantly in vaccinated and non-vaccinated T2D patients with regard to ICU admission.

Similar to in-hospital mortality and intensive care admission, patients who required endotracheal intubation had significantly lower levels of anti-SARS-CoV-2 spike antibodies than those who did not (BAU/ml; diabetic mean 210, 0–592 vs. 1042, 897–1186, *p* = 0.046; non-diabetic 270, 1–539 vs. 957, 879–1035, *p* < 0.001; non-diabetic, matched 148, 0–460 vs. 887, 734–1039, *p* < 0.001).

Finally, lower anti-SARS-CoV-2-spike antibody levels were indicative of oxygen administration across all subgroups except non-vaccinated T2D patients (BAU/ml; diabetic mean 735, 574–897 vs. 1474, 1234–1715, *p* < 0.001; non-diabetic 638, 538–737 vs. 1186, 1078–1294, *p* < 0.001; non-diabetic, matched 608, 440–776 vs. 1166, 918–1414, *p* < 0.001).

**Figure 2:**
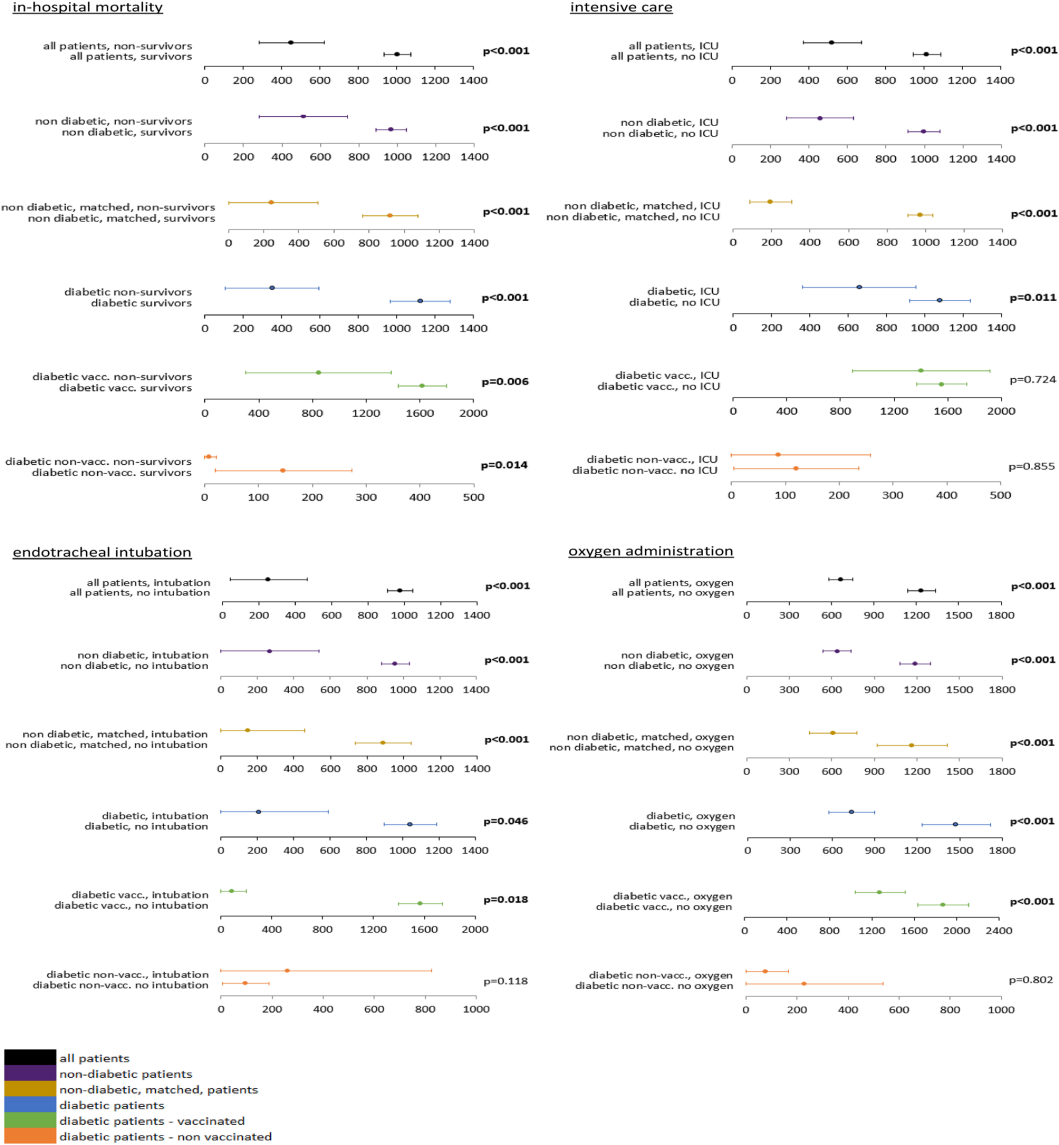
Anti-SARS-CoV-2-spike levels in BAU/ml for in-hospital mortality, admission to intensive care, endotracheal intubation and oxygen administration by diabetes and vaccination status (means ± 95% confidence intervals). vacc. vaccinated, non-vacc. non vaccinated, bold print -statistically significant.

Figure 2 shows antibody levels by outcome, stratified by patient group and vaccination status.

### Anti-SARS-CoV-2-spike antibodies and risk

Next, we aimed to quantify the risk associated with lower anti-SARS-CoV-2 antibodies in diabetic, non-diabetic and matched, non-diabetic patients. To that end, we built multiple logistic regression models for our endpoints in-hospital mortality, intensive care, endotracheal intubation and oxygen administration. We further included Cox proportional hazard models for in-hospital mortality in order to provide a second measure of risk. To facilitate interpretation of the results and improve comparability we provide risk measures for both the continuous variable in steps of 100BAU/ml and after z-score normalization. All models were then adjusted for potential confounders, including age, obesity and SARS-CoV-2 variant. Both unadjusted and adjusted odds ratios and hazard ratios are reported to show in how far these covariates affect risk of outcome.

**Figure 3:**
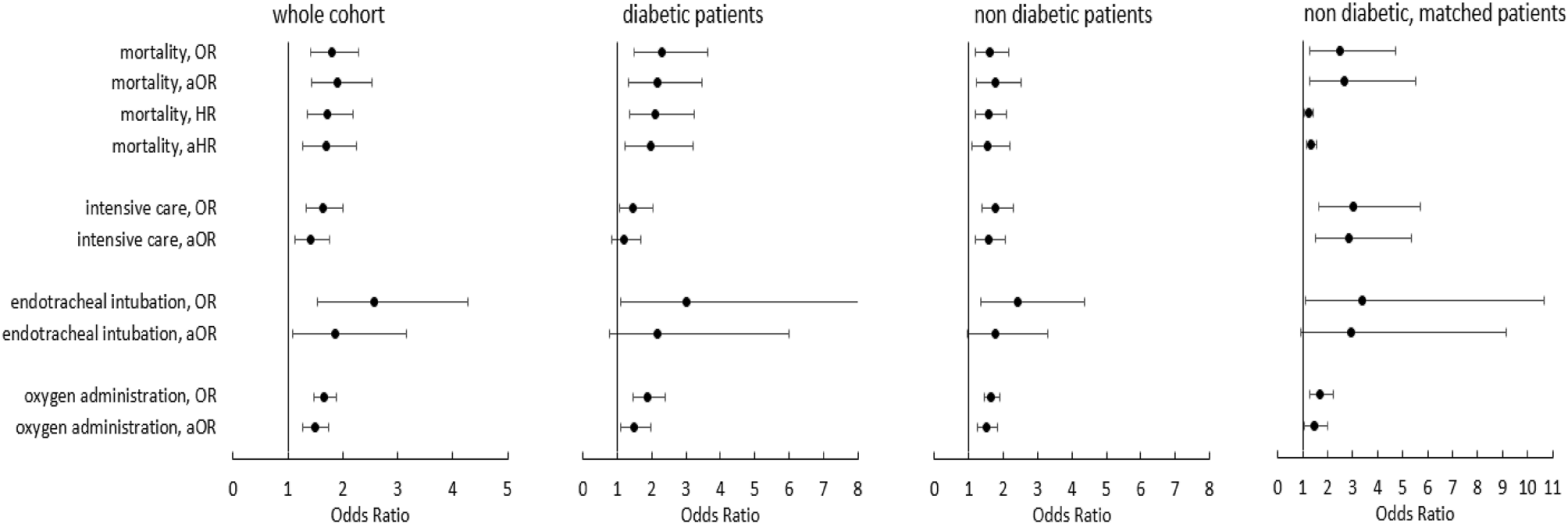
Odds ratios and adjusted odds ratios for each outcome in the whole cohort, in patients with type 2 diabetes, in non-diabetic patients and in matched, non-diabetic patients by anti-SARS-CoV-2 spike antibodies after z-score normalization. Hazard ratios and adjusted hazard ratios are given for mortality. Adjusted odds ratios are adjusted for age, obesity and SARS-CoV-2-variant.

Estimated risks for each outcome and patient group are outlined in supplemental table S1. A graphic representation of the results is provided in Fig. 3.

#### In-hospital mortality

With regard to the whole study population, risk of in-hospital mortality was 1.7 times higher for each decrement by standard deviation of anti-SARS-CoV-2 antibody levels (HR 1.721, 95% CI 1.355–2.188, *p* < 0.001; aHR 1.695, 95% CI 1.280–2.242, *p* < 0.001).

In T2D patients, risk of death was 2.1 times higher with each decrease by standard deviation of anti-SARS-CoV-2 antibody levels (HR 2.105, 95% CI 1.366–3.247, *p* < 0.001). This result remained stable after adjusting for age, obesity and SARS-CoV-2 variant (aHR 1.988, 95% CI 1.229–3.215, *p* = 0.005).

Non-diabetic patients had approximately 1.5 times higher risk of in-hospital mortality with decreasing anti-SARS-CoV-2 antibody levels (HR 1.563, 95% CI 1.170–2.088, *p* = 0.002, aHR 1.533, 1.080–2.179, *p* = 0.017). After propensity score matching, the risk of in-hospital mortality dropped slightly (HR 1.229, 95% CI 1.068–1.413, *p* = 0.004, aHR 1.329, 1.138–1.552, *p* < 0.001).

#### Secondary endpoints

Next, we investigated the risk of intensive care admission depending on SARS-CoV-2 antibody levels. For the whole study population, risk of intensive care admission was 1.4 times higher by each decrease in standard deviation of anti-SARS-CoV-2 antibody levels (aOR 1.405, 1.128–1.750). T2D patients also had significantly higher risks of requiring intensive care with lower antibody levels (OR 1.466, 95% CI 1.060–2.028), albeit these differences were not robust after adjusting for age, obesity and SARS-CoV-2 variant. In comparison, in non-diabetic patients, risk of intensive care admission increased by 1.7 times for each decrement in standard deviation of anti-SARS-CoV-2 antibody levels (OR 1.773, 95% CI 1.377–2.284, *p* < 0.001). This factor dropped slightly to 1.5 after adjusting for covariates (aOR 1.563, 95% CI 1.177–2.077, *p* = 0.002). After propensity score matching, the risk of intensive care admission in non-diabetic patients with lower antibody levels increased to 2.8 (aOR 2.827, 95% CI 1.493–5.351, *p* = 0.001).

We observed significantly higher risks of endotracheal intubation with decreasing anti-SARS-CoV-2 antibody levels for the whole study population, as well as in the subgroups of diabetic, non-diabetic and matched, non-diabetic patients before adjusting for potential confounders. However, with the exception of the whole study population (aOR 1.861, 95% CI 1.098–3.155, *p* = 0.021), these differences did not remain statistically significant after adjusting for age, obesity and SARS-CoV-2 variant.

With regard to oxygen administration, patients across all subgroups had significantly higher risk of oxygen administration with decreasing levels of anti-SARS-CoV-2 antibody before and after adjusting for potential confounders (whole cohort aOR 1.490, 95% CI 1.279–1.737, *p* < 0.001; diabetic aOR 1.476, 95% CI 1.111–1.962, *p* = 0.007; non-diabetic aOR 1.516, 95% CI 1.262–1.822, *p* < 0.001, non-diabetic, matched aOR 1.456, 95% CI 1.064–1.993, *p* = 0.019).

#### Interaction analyses

In order to better understand how anti-SARS-CoV-2 antibody levels on hospital admission are affected by the covariates age, SARS-CoV-2 variant and obesity we conducted interaction analyses for our primary endpoint, in-hospital mortality, first in the whole cohort, second in T2D patients and third in non-diabetic patients. There was no significant interaction between anti-SARS-CoV-2 antibody levels and SARS-CoV-2 variant. We also did not see a significant interaction between obesity and anti-SARS-CoV-2 antibody levels.

In contrast, we did see a significant interaction between age and anti-SARS-CoV-2 antibody levels across the whole cohort (*p* = 0.004). With regard to the whole cohort, mean antibody levels showed two peaks, one between 10 and 20 years of age and another at 70–80 years of age, with slightly decreasing means above this age. The lowest mean antibody levels were registered in patients between 40 and 50 years of age. For our T2D patients, we had very few patients aged under 40 years. Above this level, mean antibody levels in T2D patients peaked at 60–70 years of age.

### Mortality rates stratified by renal and cardial markers

T2D patients are known to have impaired microcirculation, endothelial dysfunction and reduced respiratory function and are more prone to certain comorbidities including coronary artery disease and renal disease^5–7, 11^. We therefore aimed to investigate whether mortality rates differed by creatinine and NTproBNP levels in diabetic, non-diabetic and matched, non-diabetic patients.

Patients with elevated levels of creatinine had higher mortality rates than those with normal levels across the whole cohort, non-diabetic and non diabetic matched patients (14.9% vs. 5.4%, *p* < 0.001; 12.8% vs. 4.4%, *p* < 0.001; 16.8% vs. 7.9%, *p* = 0.045). Comparable results were observed for patients with elevated levels of NTproBNP (12.5% vs. 2.3%, *p* < 0.001; 10.4% vs. 1.6%, *p* < 0.001; 15.4 vs. 0%, *p* = 0.005). In T2D patients, there was a trend towards higher mortality rates in patients with elevated creatinine (19.0% vs. 10.9%) or NTproBNP levels (18.0% vs. 6.8%) compared to patients with normal levels that did not reach statistical significance.

Among patients with elevated levels of NTproBNP, T2D patients had higher mortality rates than non-diabetic patients (18.0% vs. 10.4%, *p* = 0.005). In comparison to matched, non-diabetic patients, T2D patients still had slightly higher mortality rates, although no longer statistically significant (18.0% vs. 15.4%, *p* = 0.507). In patients with elevated levels of NTproBNP, anti-SARS-CoV-2 spike antibodies were significantly lower in non-survivors than in survivors (mean 356BAU/ml, 95% CI 185–528 vs. 1045BAU/ml, 95% CI 955–1136, *p* < 0.001).

Among patients with elevated levels of creatinine, there was no statistically significant difference in mortality between diabetic and non-diabetic patients. Low anti-SARS-CoV-2 spike antibodies continued to be strongly associated with higher in-hospital mortality in patients with elevated creatinine levels (mean 342BAU/ml, 95% CI 143–241 vs. 1050BAU/ml, 95% CI 935–1164, *p* < 0.001).

## Discussion

In this prospective, multicenter cohort study on 1152 hospitalized patients with COVID-19, we show for the first time that low anti-SARS-CoV-2 spike antibodies on hospital admission are associated with higher rates of in-hospital mortality, admission to intensive care units, endotracheal intubation and oxygen administration in T2D patients.

A correlate of protection against negative outcomes and mortality in COVID-19 may help identify patients at high risk of severe courses and inform decisions on booster vaccinations^9^. We previously reported that anti-SARS-CoV-2 spike antibodies are a strong predictor for in-hospital mortality in the general population^10^. However, due to the already elevated risk of negative outcomes, having a correlate of protection is particularly pressing in high risk subgroups like T2D patients.

Previous studies reported increased risk of hospitalization for respiratory infections unrelated to COVID-19^12^, higher rates of hospitalization for infection^13, 14^ and infection related mortality rates^15^ in T2D patients.

With regard to COVID-19, several large population based studies have reported increased risks of negative outcome in T2D patients. For instance, a nationwide Swedish cohort study found that T2D patients had a two-fold increased risk of hospitalization^16^. In addition, population based studies in Scotland^17^ and Korea^18^ described increased risks of critical care and intensive care admission as well as higher rates of ventilation and oxygen requirement^19^ in T2D patients.

T2D patients are thought to be more susceptible to severe courses of COVID-19 due to a variety of factors, including hypercoagulation, increased baseline inflammation, reduced respiratory function, endothelial dysfunction and a higher prevalence of comorbidities^5–7, 11^.

Higher mortality rates may in part be caused by increased rates of hyperglycaemia^15, 20^. Increased glucose levels in monocytes of T2D patients promote viral intracellular replication in vitro and enhance expression of pro-inflammatory cytokines that have been linked to the COVID-19 cytokine storm^12, 21^. Mechanisms that might impair immune responses in T2D patients and increase susceptibility to infections further include a reduced T-cell response, decreased secretion of IL-1 and IL-6 and reduced neutrophil function^12, 22–24^.

Other areas also report higher mortality rates in diabetic compared to non-diabetic patients. In a meta-analysis of close to one million adults, the presence of diabetes roughly doubled occlusive vascular mortality risk in men and tripled risk in women independent of other major vascular risk factors^25^. A large epidemiological study from England also reported a persistent gap in mortality between diabetic and non-diabetic patients, despite an overall decline in cardiovascular related death rates^26^.

On the other hand, the higher prevalence of serious comorbidities, including cardiovascular and renal diseases, in T2D patients could potentially influence outcome and thus confound mortality rates in unmatched comparisons between diabetic and non-diabetic patients.

In our study population, T2D patients were significantly older, had higher BMIs and had significantly higher rates of hypertension, coronary artery disease, heart failure, cerebrovascular disease and renal disease. While T2D patients showed elevated mortality rates in comparison to unmatched non-diabetic patients, this difference was markedly reduced when comparing to matched, non-diabetic patients. Accordingly, after stratifying patients by elevated creatinine or NTproBNP levels, we did not see a significant difference in mortality rates between diabetic and matched, non-diabetic patients. This suggests that diabetic patients may not be predisposed to higher mortality risks in COVID-19 due to diabetes per se but due to a range of other factors including higher age, higher BMIs and a higher prevalence of serious comorbidities. Nevertheless, these characteristics define a high risk patient subgroup that had reduced mortality risks with higher levels of anti-SARS-CoV-2 spike antibodies on hospital admission. This is also supported by better outcomes in vaccinated T2D patients than in non-vaccinated T2D patients in this cohort. All in all, our data point to an elevated risk in diabetes due to comorbidities rather than due to the diabetic state.

Previous data suggests that the production of anti-SARS-CoV-2 antibodies in patients with type 2 diabetes is dependent on glycaemic control^27^. One year after vaccination, patients with poor glycaemic control (HbA1c ≥ 7%) showed lower antibody levels and a higher incidence of breakthrough infections than patients with good glycaemic control (HbA1c < 7%). In addition, elevated HbA1c levels have been linked to an increased risk of COVID-19 mortality^28^.

While we also observed a trend towards higher antibodies in patients with better glycaemic control, these differences were not statistically significant in our study population. Further, patients with poor glycaemic control had slightly higher mortality rates than patients with good glycaemic control, albeit not a statistically significant level. Accordingly, adjusting for the potential confounder HbA1c did not affect statistical significance in our logistic regression models.

This study has several strengths. First, the high recruitment rate of this study minimizes risk of selection bias and thus constitutes one of its principal strengths. Second, this study reports a hard primary endpoint, i.e. in-hospital mortality, which does not depend on subjective clinical patient assessment and is therefore less prone to assessment bias than softer clinical endpoints^29^. Although our clinical, secondary endpoints are subject to some assessment bias, they nevertheless provide relevant information on severity and outcome in non-lethal cases of COVID-19.

Third, in order to improve comparability between diabetic and non-diabetic patients, we conducted propensity score matching and reported results both for unmatched and matched patient subgroups. Fourth, all regression models were adjusted for multiple potential confounders that are known risk factors for severe courses and higher mortality in COVID-19 including age, SARS-CoV-2 variant and obesity^30–33^.

Regarding limitations, this study was designed with a focus on hospitalized patients, making its results less applicable for outpatients. However, as severe cases of COVID-19 are likely to be treated in a hospital, it is critical to investigate this patient group. While we do not dispose of follow up data after discharge, severe cases of COVID-19, or any acute infection, are more likely to succumb during hospitalisation than after having been discharged. We therefore deemed it important to focus on the critical, acute stages of the infection.

Considering the notable increase in severe COVID-19 and mortality in T2D patients with decreasing levels of antibodies, providing regular booster vaccinations and verifying vaccine effectiveness in patients with additional risk factors is critical for protecting this vulnerable patient group. As antibody production in response to infection or vaccination is highly variable^34–36^ and antibody levels decline over time^37, 38^, future strategies for booster vaccinations in high risk populations may include measuring antibody levels to ensure patients with type 2 diabetes continue to be protected against adverse outcomes.

Concurrently, measuring anti-SARS-CoV-2 spike antibodies in COVID-19 patients with T2D could flag individuals at high risk of severe courses and COVID-19 related mortality and allow for a timely adjustment of therapy.

SARS-CoV-2 continues to affect millions of patients, with high risk subgroups like diabetic and obese patients being among the most severely affected. As SARS-CoV-2 variants with new mechanisms to evade immune responses are likely to emerge, further studies will be needed to determine whether anti-SARS-CoV-2 spike antibodies remain useful as a correlate of protection in these variants.

In conclusion our data suggest, that anti-SARS-CoV-2 spike antibody levels on hospital admission are inversely associated with oxygen administration, endotracheal intubation, admission to intensive care and in-hospital mortality in hospitalized, type 2 diabetic patients with COVID-19.

## Methods

### Study design and participants

The design of this study has previously been reported elsewhere^10^. In this prospective, multicenter cohort study, we consecutively enrolled hospitalized patients from five Austrian hospitals, who were admitted between August 1st, 2021 and April 10th, 2022.

Eligibility criteria required a positive SARS-CoV-2 test result from a polymerase chain reaction (PCR)-based assay and the collection of a blood sample on hospital admission. Exclusion criteria were previous hospitalization during the study period and ongoing hospital stay at the end of the study.

Minimum sample size was set at 465 participants, as determined by sample size calculation for logistic regression with a binary covariate, Wald’s method (alpha 0·05, two-sided, power 0·8).

### Variables

The primary endpoint of this study was in-hospital mortality from any cause. Secondary endpoints were admission to an intensive care unit, endotracheal intubation and oxygen requirement.

Patients were classified as diabetic if they had a previous diagnosis of type 2 diabetes according to ICD-10 codes, or if HbA1c levels obtained from patient files were above 6·4%. Patients with a BMI of 30 or above were considered obese.

Patients who had received one dose of an accepted single-dose vaccine or two doses of an accepted two-dose series against SARS-CoV-2 were classified as vaccinated.

Predefined covariates were selected based on known risk factors that predispose to severe courses and higher mortality in COVID-19. One of the main risk factors in COVID-19 is age, likely due to an increase in underlying conditions and general frailty^30^. In addition, type 2 diabetes and obesity have been shown to elevate mortality risk based on a combination of different factors including inflammation, hypercoagulation and mechanical obstruction^5, 39^. Mortality rates also vary by SARS-CoV-2 variant, with higher mortality rates having been documented for the Delta variant in comparison to the Omicron variant^31, 32^.

Laboratory parameters that were used as covariates were anti-SARS-CoV-2 spike antibodies, serum creatinine and NT-proBNP levels. Elevated creatinine levels were defined as > 1.2 mg/dl in men and > 0.9 mg/dl in women, elevated NTproBNP levels were defined as > 125 pg/ml.

### Data sources and measurements

All laboratory parameters were measured on Roche Cobas 6000 or Cobas 8000 systems, using the Elecsys Anti-SARS-CoV-2 S assay for antibodies against spike protein, the Creatinine Jaffé Gen.2 assay for creatinine and the Elecsys proBNP II assay for NT-proBNP.

Clinical data were obtained from patient files and comprised general information such as age, gender and body mass index (BMI), COVID-related information including COVID-19 variant and PCR-derived cycle threshold values, vaccination status and type of vaccine, as well as information regarding hospital admission including reason for hospitalization, main diagnosis, symptoms at admission, oxygen requirement (none/without endotracheal intubation/with endotracheal intubation), duration of hospital stay, admission to and duration on intensive care unit (ICU) and in-hospital survival. In T2D patients, HbA1c was obtained from patient files from measurements conducted either after hospitalisation or in the 3 months preceding hospitalisation. All HbA1c measurements were conducted using high performance liquid chromatography (HPLC) on Sysmex Tosoh G8 systems.

### Statistical methods

Statistical analyses were conducted using the IBM Statistical Package for the Social Sciences (SPSS), version 28 (IBM, Armonk, NY, USA). We reported baseline statistical characteristics, including frequencies, percentages, means, 95% confidence intervals for means, medians, standard deviations and interquartile ranges. Statistical significance was determined by Mann Whitney U tests for continuous and chi square tests for categorical variables. Two sided *p*-values of < 0.05 were considered statistically significant.

Due to significant differences in patient characteristics between diabetic and non-diabetic patients, we conducted propensity score matching to enhance comparability between the groups. Covariate selection for propensity matching was based on potential relations to outcome and/or diabetes diagnosis and on significant differences between T2D patients and control group. Groups were matched by age, BMI, and presence of hypertension, coronary artery disease, heart failure, chronic obstructive pulmonary disease, renal disease and cerebrovascular disease. Matching was conducted with a maximum difference in propensity score of 0.1 and replacement. Next, propensity scores of both groups were tested for common support and overlap was deemed satisfactory. We then evaluated balance by comparing standardized mean differences of covariates. The standardized mean difference across all covariates was 14.5% before and 6.9% after propensity score matching. Balance was achieved in all covariates except for BMI, hypertension and renal disease, although the difference between these groups was markedly reduced by propensity score matching (compare Table 1).

To evaluate the risk associated with lower anti-SARS-CoV-2 spike antibody levels, we built logistic regression models for the whole cohort and the subgroups of diabetic, non-diabetic and matched, non-diabetic patients for each endpoint (mortality/ intensive care treatment/ endotracheal intubation/ oxygen requirement). To ensure validity, basic assumptions for logistic regression analysis were tested and met. In particular, we tested for lack of duplicate entries (independence of errors), linear relationships between continuous variables and the logit transformation of the dependent variable (Box-Tidwell test), absence of multicollinearity and lack of strongly influential outliers. Regression models were calculated using a direct model-building approach, with all independent variables being added simultaneously and with equal importance. Primary and secondary endpoints (in-hospital mortality/ intensive care treatment/ endotracheal intubation/ oxygen requirement) were entered as dichotomous, dependent variable whereas predefined covariates were set as independent variables. Odds ratios were reported with 95% confidence intervals.

As a second measure of risk, we used a Cox proportional hazard model to provide hazard ratios for our primary endpoint, in-hospital mortality. The proportional hazard model was built as described in the previous paragraph, with time to event measured in days from hospital admission. As is customary, hazard ratios for in-hospital mortality were reported with a 95% confidence interval.

The described models were then adjusted to compensate for potential confounding by the covariates age, obesity and SARS-CoV-2 variant. To avoid overfitting of the regression models, we verified having sufficient event counts in the whole cohort and the subgroups of diabetic, non-diabetic and matched, non-diabetic patients, before adjusting for potential confounders. To improve comparability, results from logistic regression analysis and the Cox proportional hazard model are reported both for spike antibodies in steps of 100BAU/ml and after z-score-normalisation of the parameter.

In order to improve our understanding of how anti-SARS-CoV-2 antibody levels on hospital admission are affected by the covariates age, SARS-CoV-2 variant and obesity, we conducted interaction analyses for our primary endpoint, in-hospital mortality, across the whole cohort as well as in diabetic patients and in non-diabetic patients.

To test the robustness of our findings, we rebuilt the regression models described above while applying bootstrapping with 2000 samples first for the whole cohort, second for diabetic patients, third for non-diabetic patients, and fourth for matched, non-diabetic patients. Hosmer–Lemeshow tests were applied to test goodness of fit.

### Ethical approval and informed consent

The local Institutional Review Board, Ethikkommission Vorarlberg, Roemerstrasse 15, A-6901 Bregenz, approved the protocol and waived the need to obtain informed consent from the study participants. The study was carried out in accordance with the Declaration of Helsinki of 1975 (revised 2013) and Good Clinical Research Practice.

### Supplementary Information

Supplementary Table S1.

## Data Availability

As personal individual information is included in the dataset, the data pertaining to this investigation is not publically available to protect study participant privacy. However, an anonymised version will be shared upon reasonable request to the corresponding author.

## Abbreviations

COVID-19: Coronavirus disease 2019
SARS-CoV-2: Severe acute respiratory syndrome coronavirus 2
PCR: Polymerase chain reaction
ICU: Intensive care unit
OR, aOR, HR: Odds ratio, adjusted odds ratio, hazard ratio
CI: Confidence interval
LDL-C: Low density lipoprotein
HDL-C: High density lipoprotein
BMI: Body mass index
COPD: Chronic obstructive pulmonary disease
CAD: Coronary artery disease
TIA: Transient ischemic attack
CVD: Cerebrovascular disease
BAU/ml: Binding antibody units/ml

## References

[1] Li R (2023). Global diabetes prevalence in COVID-19 patients and contribution to COVID-19- related severity and mortality: A systematic review and meta-analysis. Diabetes Care.

[2] Edqvist J (2023). Severe COVID-19 Infection in type 1 and type 2 diabetes during the first three waves in Sweden. Diabetes Care.

[3] Schäfer E (2022). Course of disease and risk factors for hospitalization in outpatients with a SARS-CoV-2 infection. Sci. Rep..

[4] Chenchula S (2023). Global prevalence and effect of comorbidities and smoking status on severity and mortality of COVID-19 in association with age and gender: A systematic review, meta-analysis and meta-regression. Sci. Rep..

[5] Steenblock C (2021). COVID-19 and metabolic disease: Mechanisms and clinical management. Lancet Diabetes Endocrinol..

[6] Durrington P (2023). Blood lipids after COVID-19 infection. Lancet Diabetes Endocrinol..

[7] Ayres JS (2020). A metabolic handbook for the COVID-19 pandemic. Nat. Metab..

[8] Ekpor E, Akyirem S (2023). Global acceptance of COVID-19 vaccine among persons with diabetes: A systematic review and meta-analysis. Diabetes Res. Clin. Pract..

[9] Krammer F (2021). A correlate of protection for SARS-CoV-2 vaccines is urgently needed. Nat. Med..

[10] Mink S (2023). Evaluation of SARS-CoV-2 antibody levels on hospital admission as a correlate of protection against mortality. J. Intern. Med..

[11] Strain WD, Paldánius PM (2018). Diabetes, cardiovascular disease and the microcirculation. Cardiovasc. Diabetol..

[12] Tomic D, Shaw JE, Magliano DJ (2022). The burden and risks of emerging complications of diabetes mellitus. Nat. Rev. Endocrinol..

[13] Fang M (2021). Diabetes and the risk of hospitalisation for infection: The atherosclerosis risk in communities (ARIC) study. Diabetologia.

[14] Luk AOY (2021). Temporal trends in rates of infection-related hospitalisations in Hong Kong people with and without diabetes, 2001–2016: A retrospective study. Diabetologia.

[15] Magliano DJ (2015). Excess risk of dying from infectious causes in those with type 1 and type 2 diabetes. Diabetes Care.

[16] Rawshani A (2021). Severe COVID-19 in people with type 1 and type 2 diabetes in Sweden: A nationwide retrospective cohort study. Lancet Region Health Eur..

[17] McGurnaghan SJ (2021). Risks of and risk factors for COVID-19 disease in people with diabetes: A cohort study of the total population of Scotland. Lancet Diabetes Endocrinol..

[18] You JH (2020). Clinical outcomes of COVID-19 patients with type 2 diabetes: A population-based study in Korea. Endocrinol. Metab. (Seoul, Korea).

[19] Moon SJ (2020). Independent impact of diabetes on the severity of coronavirus disease 2019 in 5307 patients in South Korea: A nationwide cohort study. Diabetes Metab. J..

[20] Lombardi A, Agarwal S, Schechter C, Tomer Y (2022). In-hospital hyperglycemia is associated with worse outcomes in patients admitted with COVID-19. Diabetes Care.

[21] Codo AC (2020). Elevated glucose levels favor SARS-CoV-2 infection and monocyte response through a HIF-1α/glycolysis-dependent axis. Cell Metab..

[22] Geerlings SE, Hoepelman AI (1999). Immune dysfunction in patients with diabetes mellitus (DM). FEMS Immunol. Med. Microbiol..

[23] Velazquez-Salinas L, Verdugo-Rodriguez A, Rodriguez LL, Borca MV (2019). The role of interleukin 6 during viral infections. Front. Microbiol..

[24] Joshi N, Caputo GM, Weitekamp MR, Karchmer AW (1999). Infections in patients with diabetes mellitus. N. Engl. J. Med..

[25] Gnatiuc L (2018). Sex-specific relevance of diabetes to occlusive vascular and other mortality: A collaborative meta-analysis of individual data from 980 793 adults from 68 prospective studies. Lancet Diabetes Endocrinol..

[26] Pearson-Stuttard J (2021). Trends in predominant causes of death in individuals with and without diabetes in England from 2001 to 2018: An epidemiological analysis of linked primary care records. Lancet Diabetes Endocrinol..

[27] Marfella R (2022). Glycaemic control is associated with SARS-CoV-2 breakthrough infections in vaccinated patients with type 2 diabetes. Nat. Commun..

[28] Prattichizzo F, de Candia P, Nicolucci A, Ceriello A (2022). Elevated HbA1c levels in pre-Covid-19 infection increases the risk of mortality: A sistematic review and meta-analysis. Diabetes/Metab. Res. Rev..

[29] Drexel H (2020). The age of randomized clinical trials: Three important aspects of randomized clinical trials in cardiovascular pharmacotherapy with examples from lipid and diabetes trials. Eur. Heart J. Cardiovasc. Pharmacother..

[30] Clark A (2020). Global, regional, and national estimates of the population at increased risk of severe COVID-19 due to underlying health conditions in 2020: A modelling study. Lancet Glob. Health.

[31] Nyberg T (2022). Comparative analysis of the risks of hospitalisation and death associated with SARS-CoV-2 omicron (B11529) and delta (B16172) variants in England: A cohort study. Lancet (London, England).

[32] Webster HH (2022). Hospitalisation and mortality risk of SARS-COV-2 variant omicron sub-lineage BA.2 compared to BA.1 in England. Nat. Commun..

[33] Kwok S (2020). Obesity: A critical risk factor in the COVID-19 pandemic. Clin. Obes..

[34] Smoot K (2022). Persistence and protective potential of SARS-CoV-2 antibody levels after COVID-19 vaccination in a West Virginia nursing home cohort. JAMA Netw. Open.

[35] Rijkers G (2020). Differences in antibody kinetics and functionality between severe and mild severe acute respiratory syndrome coronavirus 2 infections. J. Infect. Dis..

[36] Milani GP (2020). Serological follow-up of SARS-CoV-2 asymptomatic subjects. Sci. Rep..

[37] Dan JM (2021). Immunological memory to SARS-CoV-2 assessed for up to 8 months after infection. Science (New York, N.Y.).

[38] Seow J (2020). Longitudinal observation and decline of neutralizing antibody responses in the 3 months following SARS-CoV-2 infection in humans. Nat. Microbiol..

[39] Vasbinder A (2022). Inflammation, hyperglycemia, and adverse outcomes in individuals with diabetes mellitus hospitalized for COVID-19. Diabetes Care.

