# Supplementary material for: Anti-SARS-CoV-2 antibody levels predict outcome in COVID-19 patients with type 2 diabetes: a prospective cohort study
Source: Sci Rep. 2023 Oct 26;13:18326. doi: 10.1038/s41598-023-45700-4 (PMC10603091; doi:10.1038/s41598-023-45700-4)
Supplement: Supplementary file 1 — Supplementary Table S1. [file 41598_2023_45700_MOESM1_ESM.docx]

|  | **OR, 95%CI** | **p-value** | **aOR, 95%CI** | **p-value** | **OR z-scores, 95%CI** | **p-value** | **aOR z-scores, 95%CI** | **p-value** |
| --- | --- | --- | --- | --- | --- | --- | --- | --- |
| **all patients** |  |  |  |  |  |  |  |  |
| mortality | 1.052, 1.030-1.075 | **<0.001** | 1.058, 1.032-1.084 | **<0.001** | 1.794, 1.405-2.289 | **<0.001** | 1.906 1.431-2.538 | **<0.001** |
| mortality, HR | 1.048, 1.027-1.071 | **<0.001** | 1.047, 1.021-1.073 | **<0.001** | 1.721, 1.355-2.188 | **<0.001** | 1.695, 1.280-2.242 | **<0.001** |
| ICU | 1.044, 1.026-1.062 | **<0.001** | 1.030, 1.010-1.050 | **0.002** | 1.641, 1.341-2.000 | **<0.001** | 1.405, 1.128-1.750 | **0.002** |
| intubation | 1.085, 1.039-1.135 | **<0.001** | 1.055, 1.008-1.105 | **0.021** | 2.571, 1.546-4.274 | **<0.001** | 1.861, 1.098-3.155 | **0.021** |
| oxygen | 1.045, 1.034-1.056 | **<0.001** | 1.035, 1.022-1.049 | **<0.001** | 1.661, 1.472-1.874 | **<0.001** | 1.490, 1.279-1.737 | **<0.001** |
|  |  |  |  |  |  |  |  |  |
| **diabetic** |  |  |  |  |  |  |  |  |
| mortality | 1.075, 1.034-1.118 | **<0.001** | 1.069, 1.026-1.114 | **0.002** | 2.309, 1.474-3.618 | **<0.001** | 2.159, 1.339-3.480 | **0.002** |
| mortality, HR | 1.067, 1.028-1.107 | **<0.001** | 1.062, 1.018-1.107 | **0.005** | 2.105, 1.366-3.247 | **<0.001** | 1.988, 1.229-3.215 | **0.005** |
| ICU | 1.034, 1.005-2.063 | **0.021** | 1.015, 0.984-1.047 | 0.346 | 1.466, 1.060-2.028 | **0.021** | 1.187, 0.831-1.694 | 0.346 |
| intubation | 1.101, 1.008-1.202 | **0.033** | 1.069, 0.979-1.168 | 0.137 | 3.016, 1.095-8.304 | **0.033** | 2.165, 0.783-5.987 | 0.137 |
| oxygen | 1.056, 1.033-1.079 | **<0.001** | 1.034, 1.009-1.060 | **0.007** | 1.863, 1.449-2.395 | **<0.001** | 1.476, 1.111-1.962 | **0.007** |
|  |  |  |  |  |  |  |  |  |
| **non-diabetic** |  |  |  |  |  |  |  |  |
| mortality | 1.042, 1.016-1.069 | **0.002** | 1.050, 1.018-1.083 | **0.002** | 1.605, 1.198-2.149 | **0.002** | 1.755, 1.225-2.514 | **0.002** |
| mortality, HR | 1.040, 1.014-1.066 | **0.002** | 1.038, 1.007-1.070 | **0.017** | 1.563, 1.170-2.088 | **0.002** | 1.533, 1.080-2.179 | **0.017** |
| ICU | 1.051, 1.028-1.074 | **<0.001** | 1.040, 1.014-1.066 | **0.002** | 1.773, 1.377-2.284 | **<0.001** | 1.563, 1.177-2.077 | **0.002** |
| intubation | 1.080, 1.026-1.137 | **0.003** | 1.051, 0.995-1.109 | 0.073 | 2.423, 1.345-4.363 | **0.003** | 1.764, 0.949-3.280 | 0.073 |
| oxygen | 1.045, 1.032-1.058 | **<0.001** | 1.037, 1.020-1.054 | **<0.001** | 1.653, 1.435-1.904 | **<0.001** | 1.516, 1.262-1.822 | **<0.001** |
|  |  |  |  |  |  |  |  |  |
| **non-diabetic, matched** |  |  |  |  |  |  |  |  |
| mortality | 1.082, 1.023-1.145 | **0.006** | 1.089, 1.022-1.160 | **0.008** | 2.481, 1.299-4.739 | **0.006** | 2.669, 1.291-5.516 | **0.008** |
| mortality, HR | 1.018, 1.006-.1031 | **0.004** | 1.025, 1.011-1.039 | **<0.001** | 1.229, 1.068-1.413 | **0.004** | 1.329, 1.138-1.552 | **<0.001** |
| ICU | 1.101, 1.043-1.163 | **<0.001** | 1.094, 1.035-1.157 | **0.001** | 3.043, 1.624-5.699 | **<0.001** | 2.827, 1.493-5.351 | **0.001** |
| intubation | 1.112, 1.008-1.228 | **0.035** | 1.098, 0.994-1.212 | 0.066 | 3.405, 1.090-10.638 | **0.035** | 2.921, 0.931-9.159 | 0.066 |
| oxygen | 1.046, 1.021-1.071 | **<0.001** | 1.033, 1.005-1.062 | **0.019** | 1.674, 1.271-2.204 | **<0.001** | 1.456, 1.064-1.993 | **0.019** |

Supplemental table S1. Odds ratios and adjusted odds ratios for each outcome for anti-SARS-CoV2-spike antibodies both in decrements of 100U/ml (left) and after z-standardisation (right). Adjusted odds ratios were adjusted for age, obesity and SARS-CoV2 variant. OR odds ratio, aOR adjusted odds ratio, HR hazard ratio, ICU intensive care unit, intubation-endotracheal intubation, oxygen- oxygen administration, bold print- statistically significant.
